# Nanosilica-Anchored Polycaprolactone/Chitosan Nanofibrous Bioscaffold to Boost Osteogenesis for Bone Tissue Engineering

**DOI:** 10.3390/molecules27248832

**Published:** 2022-12-13

**Authors:** Shengyou Ge, Xiaoyi Zhu, Chuanlong Zhang, Dongchen Jia, Wei Shang, Chao Ding, Jianping Yang, Yuanyong Feng

**Affiliations:** 1 Department of Oral and Maxillofacial Surgery, School of Stomatology and The Affiliated Hospital of Qingdao University, No. 16 Jiangsu Road, Qingdao 266003, China; 2 School of Environmental Science and Engineering, Qingdao University, No. 308 Ningxia Road, Qingdao 266071, China; 3 Longkou Traditional Chinese Medicine Hospital, Longkou 265700, China; 4 State Key Laboratory for Modification of Chemical Fibers and Polymer Materials, College of Materials Science and Engineering, Donghua University, Shanghai 201620, China

**Keywords:** silica, polycaprolactone, chitosan, bioscaffold, osteogenesis, bone tissue engineering

## Abstract

The strategy of incorporating bioactive inorganic nanomaterials without side effects as osteoinductive supplements is promising for bone regeneration. In this work, a novel biomass nanofibrous scaffold synthesized by electrospinning silica (SiO_2_) nanoparticles into polycaprolactone/chitosan (PCL/CS) nanofibers was reported for bone tissue engineering. The nanosilica-anchored PCL/CS nanofibrous bioscaffold (PCL/CS/SiO_2_) exhibited an interlinked continuous fibers framework with SiO_2_ nanoparticles embedded in the fibers. Compact bone-derived cells (CBDCs), the stem cells derived from the bone cortex of the mouse, were seeded to the nanofibrous bioscaffolds. Scanning electron microscopy and cell counting were used to observe the cell adhesion. The Counting Kit-8 (CCK-8) assay was used. Alkaline phosphatase (ALP), Alizarin red staining, real-time Polymerase Chain Reaction and Western blot tests were performed to confirm the osteogenesis of the CBDCs on the bioscaffolds. The research results demonstrated that the mechanical property of the PCL together with the antibacterial and hydrophilic properties of the CS are conducive to promoting cell adhesion, growth, migration, proliferation and differentiation. SiO_2_ nanoparticles, serving as bone induction factors, effectively promote the osteoblast differentiation and bone regeneration. This novel SiO_2_-anchored nanofibrous bioscaffold with superior bone induction activity provides a better way for bone tissue regeneration.

## 1. Introduction

The regeneration and repair of bone defects are clinical key issues that need to be addressed [1]. In the bone graft regeneration technology, an autograft transplantation technique is a priority selection due to the good biological activity and no immune rejection. However, complications such as tissue infection, necrosis, etc., hinder its widespread application [2,3]. The bone allograft is an alternative strategy but is facing limited application in clinics due to the high cost, disease transmission risk and poor host immune response [4,5].

The development of a synthetic bone graft is an advocated strategy to address the above issues [6]. The emergence of tissue engineering is to inoculate cells on a biological scaffold, where cells can adhere and grow, and finally regenerate and repair bone defects [7]. The key factor of tissue engineering technology is the characteristics of the scaffold materials. An ideal scaffold material should have suitable mechanical properties, biocompatibility, a three-dimensional structure and can promote bone tissue growth [8]. Hydrogel [9], 3D-printed scaffolds [10], nanofibers, nanoparticles and nanofilms are promising scaffolds for tissue engineering [11], which have attracted much attention due to their high surface-area-to-volume ratio, high tensile strength, high permeability associated with high biocompatibility and other characteristics [12]. Moreover, nanofibrous scaffolds exhibit a nanofibers network which can be integrated into biological systems by mimicking the environment of a natural extracellular matrix [13]. Various methods such as electrospinning [14,15], template polymerization [16] and the self-assembly method [17,18] have been used to synthesize nanofibers. Among these, nanofibers prepared by the electrospinning technique exhibit a three-dimensional porous structure and the electrospun nanofibers display similar diameters to the cells [8]. In addition, the obtained electrospun nanofibrous scaffolds loading seed cells and bone induction factors can promote cell adhesion, proliferation and differentiation and simulate an extracellular structure in vitro, which exhibit its unique advantages for a primary cell culture and subculture [19].

Biomass polymers, i.e., polylactic acid [20], collagen [21], chitosan (CS) [22], polycaprolactone (PCL) [23], gelatin [24] and bovine serum albumin [25], have been investigated as the raw materials for electrospinning nanofibrous bioscaffolds in tissue engineering. The CS has a wide application prospect as a scaffold material due to the excellent biocompatibility, antibacterial property, biodegradability and low toxicity. However, the mechanical property and poor plasticity make it difficult to maintain the structural integrity [5]. The PCL presents an excellent mechanical property and processability but a high hydrophobicity and low degradation rate [26,27]. Thus, electrospun nanofibrous scaffolds comprised of CS and PCL are expected to exhibit not only good mechanical properties but also favorable biological activity. Saderi et al. [28] added the natural hydrophilic antimicrobial CS to a PCL scaffold to resolve the poor hydrophilic and antimicrobial properties of the PCL for tissue repair scaffolds. Mahoney et al. [29] studied the influence of the PCL/CS ratio on the performance of tissue repair scaffolds. It is concluded that PCL/CS with the mass ratio of 7:3 presents both mechanical properties and hydrophilic and antibacterial properties, which are more conducive to promoting tissue repair.

The incorporation of the induction factors of bone formation into the scaffolds is an effective strategy to improve the mesenchymal stem cell (MSC) recruiting [19]. Silicon (Si) is an essential element for bone metabolism which triggers the osteogenic differentiation of MSC [30,31]. Silica modification, due to its osteoinduction activity, has become a potential alternative for silicification collagen scaffolds, and the osteogenesis silica composites have been developed [32]. Wang et al. [32] reported the resultant nanosilica-collagen (nSC) composites which possess a native-bone-like porous structure and a nanosilica coating. The osteoinductivity of the nSC scaffolds is strongly dependent on the surface roughness and Si content in the silica coating. Wang et al. [33] fabricated electrospun nanosilicates-doped nanofibers for bone tissue engineering. The results of histological and immunohistochemical assessments revealed that the nanosilicates-enriched nanofibers had a significant potential of ectopic bone formation in vivo, while the pure PCL samples only induced limited osteogenic cues.

**Scheme 1:**
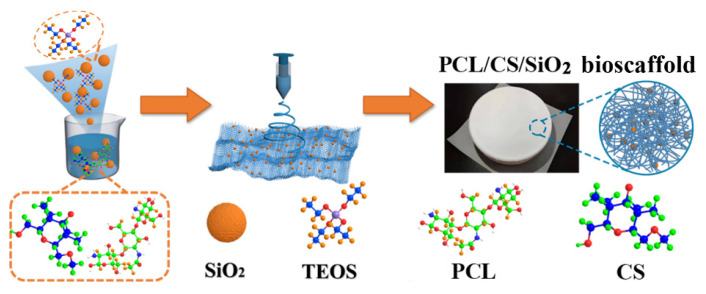
The schematic illustration of PCL/CS/SiO_2_ bioscaffold.

Herein, a novel nanofibrous bioscaffold consisting of electrospun PCL/CS nanofibers-encapsulated silica (SiO_2_) nanoparticles was demonstrated for bone regeneration tissue engineering (Scheme 1). The PCL has good biocompatibility and provides mechanical strength and reduces the inflammation effect [34,35]. CS has good biocompatibility, degradation and absorption, a strong plastic mechanical property and can support the adhesion and differentiation of bone marrow MSCs. SiO_2_ nanoparticles, a bioactive inorganic nanomaterial without side effects, are selected as the induction factor of the bone formation due to the osteoinduction activity. In addition, the polymeric nanofibers loaded with SiO_2_ nanoparticles are capable of improving physical and mechanical properties. In addition, the SiO_2_ surface, which is rich in silanol groups, can effectively improve the surface hydrophilic property and form nano-active silicate materials in body fluids to combine with natural bone tissue. The released Si ions can promote the growth and proliferation of MSCs and the expression of osteogenesis genes. The synthesized PCL/CS/SiO_2_ bioscaffolds demonstrate high potential for clinical translation in the treatment of bone defects in bone tissue repair engineering.

## 2. Results and Discussion

### 2.1. Structural and Morphology Characterization of the PCL/CS and PCL/Cs/SiO_2_ Bioscaffolds

**Figure 1:**
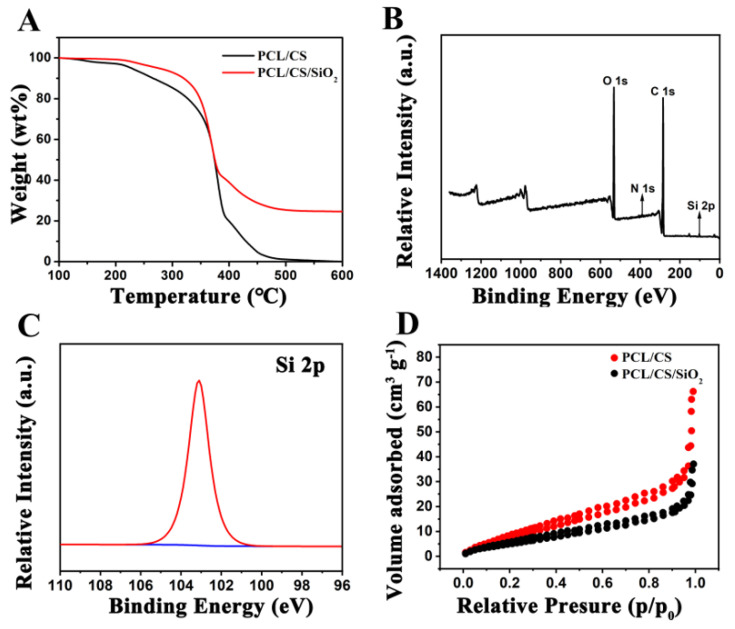
TG curves (**A**) of PCL/CS and PCL/CS/SiO_2_ bioscaffolds, XPS spectra (**B**) and Si 2p spectrum (**C**) of PCL/CS/SiO_2_ bioscaffold, and N_2_ adsorption–desorption isotherms (**D**) of PCL/CS and PCL/CS/SiO_2_ bioscaffolds.

A thermogravimetric analysis (TGA) was tested to confirm the SiO_2_ content in the electrospun bioscaffolds. Figure 1A shows the TG curves of the PCL/CS and PCL/CS/SiO_2_ bioscaffolds. The weight loss that occurred from 100 to 500 °C was ascribed to the thermal decomposition of the PCL and CS. According to the TG curves, the content of the SiO_2_ can be roughly calculated to be around 24.9% in the PCL/CS/SiO_2_. The DSC heating curve of the PCL/CS/SiO_2_ bioscaffold is displayed in Figure S1. The melting endothermic peak occurred at a lower temperature than the reported melting temperature (58.12 °C) of the PCL, indicating a low crystallization caused by the addition of SiO_2_.

An X-ray photoelectron spectroscopy (XPS) measurement was conducted to confirm the elemental composition and the valence states in the composite. Figure 1B shows the whole spectrum of the PCL/CS/SiO_2_, indicating the coexistence of Si, C and O elements. The spectrum of Si 2p in Figure 1C shows that the peak located at 103 eV is attributed to the Si-O bonds which are derived from SiO_2_ nanoparticles [36]. In addition, the spectra of C 1s and O 1s are shown in Figure S2. The FTIR spectra of the PCL/CS and PCL/CS/SiO_2_ bioscaffolds are shown in Figure S3. The peaks at around 2945 and 2864 cm^−1^ can be ascribed to the stretching vibrations of -CH_2_ groups. The C=O and C–O–C stretching vibrational peaks of the PCL appeared at around 1725 and 1158 cm^−1^, respectively. The characteristic absorption bands corresponding to the bending vibration peak of the N–H of the CS were observed at 1589 cm^−1^. For the PCL/CS/SiO_2_ bioscaffold, the Si–O–Si antisymmetric stretching vibration peak at 1091 cm^−1^ is due to the infrared absorption generated by the Si–O–Si bonds of the SiO_2_.

The N_2_ adsorption–desorption properties of the PCL/CS and PCL/CS/SiO_2_ were analyzed by a BET measurement, and the specific surface areas are illustrated in Table S1. The PCL/CS and PCL/CS/SiO_2_ bioscaffolds had specific surface areas of 23.94 and 37.68 m^2^ g^−1^, which are mainly due to the void space generated by the interlinked electrospun polymer fibers [37]. The N_2_ adsorption–desorption isotherm curves in Figure 1D display the bioscaffolds that had a distinct hysteresis loop, indicating the existence of a mesoporous structure attributed to the void space derived from the electrospun nanofibers. The porous bioscaffolds are conductive to a better cell-fiber entanglement and provide more cell adhesion sites to increasing cell adhesion, anchoring and growth [38]. The mechanical property of the bone tissue engineering scaffolds is important for the bio-functionality. Figure S4 displays the representative stress–strain curves of the PCL/CS and PCL/CS/SiO_2_ bioscaffolds in Figure S4. The tensile strength was about 4.98 and 6.01 MPa and the elongation at the break was 38.03 and 23.8% for the PCL/CS and PCL/CS/SiO_2_ bioscaffolds, respectively. The PCL has excellent flexibility and provides good ductility, while the CS has more charged groups leading to the high breaking strength. In addition, the introduction of SiO_2_ into the bioscaffold also leads to the increase in breaking strength.

**Figure 2:**
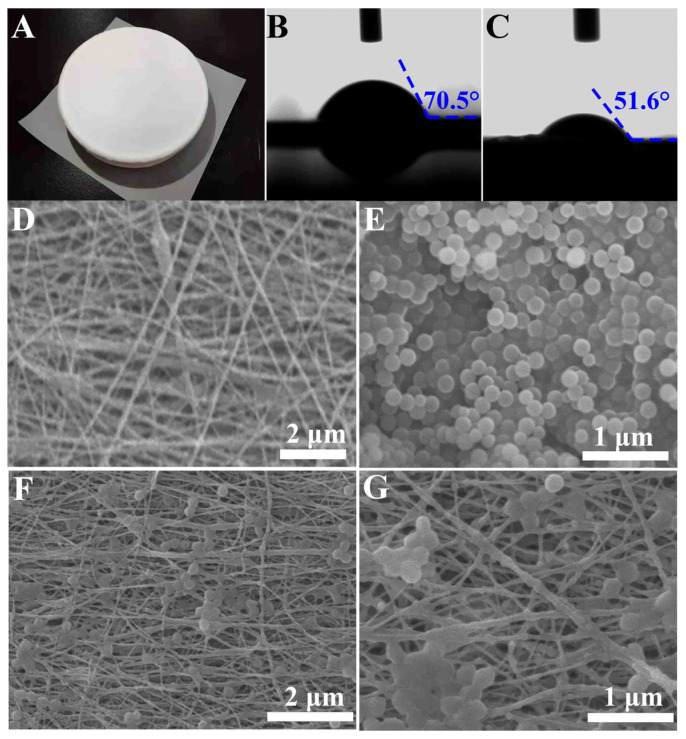
Electric photograph (**A**) of PCL/CS/SiO_2_ bioscaffold, water contact angle images of PCL/CS (**B**) and PCL/CS/SiO_2_ (**C**) bioscaffolds, SEM images of PCL/CS bioscaffold (**D**), SiO_2_ nanoparticles synthesized by the well-established Stöber method (**E**), and SEM images of PCL/CS/SiO_2_ bioscaffold (**F**) and the enlarged view (**G**).

Figure 2A shows the electric photograph of the PCL/CS/SiO_2_ bioscaffold. It can be seen that the surface of the fiber membrane was smooth, and no liquid drop was seen, confirming a superior fiber-form capability. Figure 2B,C show the images of the water contact angle analysis on the nanofibrous bioscaffolds. It is clear that the PCL/CS and PCL/CS/SiO_2_ bioscaffolds had a good hydrophilic property with an average contact angle of 70.5°and 51.6°, respectively. Due to the poor hydrophilic and antibacterial properties of the PCL, which are unfavorable for cell adhesion growth, introducing the CS content in hybrid bioscaffolds can significantly improve the hydrophilic nature of the bioscaffold. Moreover, compared with PCL/CS, the PCL/CS/SiO_2_ bioscaffold presented a better hydrophilicity property due to the hydroxyl group on the surface of the SiO_2_. The presence of SiO_2_ embedded in the interlinked fibers improves the hydrophilicity of the scaffold and provides more cell adhesion sites [39]. Figure 2D displays the electrospun PCL/CS fibers that are randomly arranged and exhibit an interlinked continuous fibers framework with the diameter of around 50~100 nm and no apparent droplets are seen. The fibers have a smooth surface and exhibit an interlaced microstructure with apparent mesopores, which can facilitate cells penetrating into the pores of the bioscaffolds. Figure 2E shows the morphology of the SiO_2_ nanoparticles prepared by the well-established Stöber method. The SiO_2_ nanoparticles exhibit a uniform sphere morphology with a diameter of around 200 nm. Figure 2F shows that PCL/CS/SiO_2_ present obvious sphere morphology nanoparticles except for the similar fibers framework with PCL/CS. It can be inferred that SiO_2_ nanoparticles were successfully anchored into the bioscaffolds. From the enlarged-view SEM image (Figure 2G), it can be seen that the SiO_2_ nanoparticles were obviously embedded in the interlinked fibers. According to the investigation on the microstructure of the bioscaffolds, it is concluded that the induction factor of the SiO_2_ nanoparticles is evenly dispersed in the nanofibers, and the bioscaffolds successfully maintain the structural integrity which is conducive to cell growth, proliferation and migration and further promote bone tissue repair.

The degradation of the biomass nanofibrous scaffold is a key factor in bone tissue engineering. Therefore, it is of great significance to study the degradation performance of the prepared bioscaffolds for guiding the modification of the nanofibers to meet the different actual needs. The water absorption expansion rate and weight loss ratio of the PCL/CS and PCL/CS/SiO_2_ bioscaffolds were measured. The water absorption was measured by immersing the PCL/CS and PCL/CS/SiO_2_ bioscaffolds in a phosphate buffer solution (PBS) for 50 h, and the results are shown in Figure S5. The water absorption ratio increased with prolonged time and reached an equilibrium level after 40 h. Compared with PCL/CS, the PCL/CS/SiO_2_ bioscaffold has higher water absorption, which may be due to the enhanced hydrophilicity of the hydroxyl group on the surface of the SiO_2_. In addition, the degradability of the PCL/CS and PCL/CS/SiO_2_ bioscaffolds was evaluated at pH = 7.4. Figure S6 displays that the weight loss ratio increased as time passed over 10 days. Compared with PCL/CS, the PCL/CS/SiO_2_ bioscaffold exhibited a higher weight loss ratio, indicating higher biodegradability.

### 2.2. Biological Tests of PCL/CS and PCL/CS/SiO_2_ Bioscaffolds

#### 2.2.1. Microphotographs and SEM Images of CBDCs on the PCL/CS and PCL/CS/SiO_2_ Bioscaffolds

**Figure 3:**
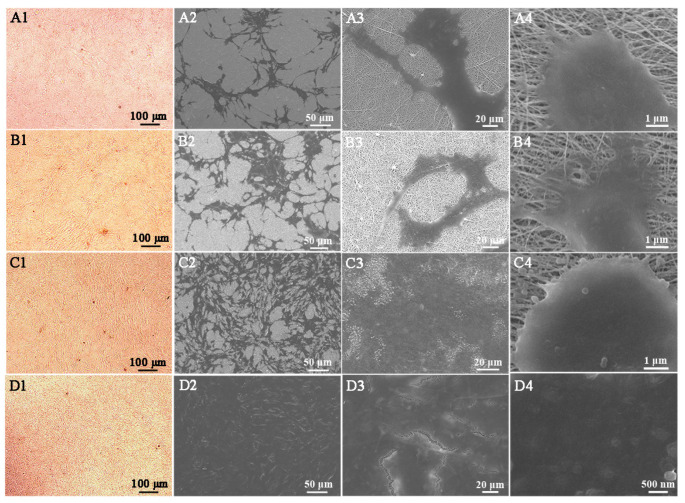
Microphotographs and the corresponding SEM images of cells on PCL/CS bioscaffold for 48 h (**A1**–**A4**), 96 h (**B1**–**B4**) and on PCL/CS/SiO_2_ bioscaffold for 48 h (**C1**–**C4**), 96 h (**D1**–**D4**).

Figure 3 shows the microphotographs and the SEM images of the CBDCs grown on the PCL/CS (Figure 3A,B) and PCL/CS/SiO_2_ (Figure 3C,D) bioscaffolds for 48 and 96 h, respectively. Compared with PCL/CS, the PCL/CS/SiO_2_ bioscaffold showed a better adherence with increased cell numbers and a better cell-fiber entanglement. Especially for the PCL/CS/SiO_2_ bioscaffold after seeding for 48 h, the CBDCs were adhered tightly and stretched well on the fibers of the bioscaffold. The cells, exhibiting a polygonal appearance on the crossed fibers, not only covered the surface but also penetrated into the pores of the fibrous bioscaffolds [40]. After 96 h of culture, the number of cells on the fibers apparently increased and the proliferating cells almost spread over the whole nanofibrous bioscaffolds. The porous electrospun nanofibrous bioscaffolds provide enough three-dimensional void space for the adhesion and proliferation of bone MSCs and deliver oxygen and nutrients through the interconnected pores to the cells on the bioscaffolds [41]. Additionally, the addition of the SiO_2_ induction factor improves the hydrophilicity of the bioscaffold, enhances the cell proliferation efficiency and reduces the risk of cell contamination, thus providing a better microenvironment for bone tissue regeneration [16].

#### 2.2.2. Cell Adhesion and Proliferation Assay with CBDCs

**Figure 4:**
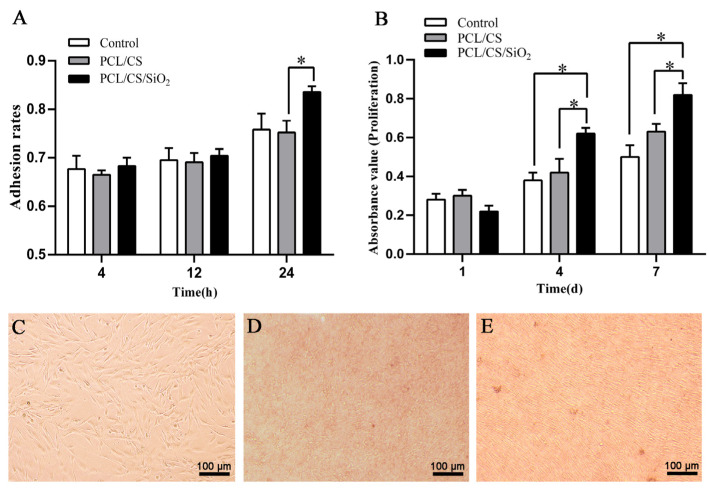
Cell adhesion and proliferation rates of CBDCs on PCL/CS and PCL/CS/SiO_2_ bioscaffolds. (**A**) CBDCs adhesion after 4, 12 and 24 h seeded on the bioscaffolds (* *p* < 0.05, *n* = 3). (**B**) CBDCs proliferation rates on PCL/CS and PCL/CS/SiO_2_ bioscaffolds at 1, 4 and 7 days (* *p* < 0.05, *n* = 3). CBDCs morphology after 4 days seeded on control (**C**), PCL/CS (**D**) and PCL/CS/SiO_2_ (**E**) bioscaffolds.

Figure 4A shows the CBDCs adhesion on the PCL/CS and PCL/CS/SiO_2_ bioscaffolds. After 4 h of culture, the cells adhesion rates on the surface of the PCL/CS and PCL/CS/SiO_2_ bioscaffolds were 66.5% and 68.3%, respectively. The cell adhesion rate of the PCL/CS and PCL/CS/SiO_2_ bioscaffolds increased to 69.1 and 70.4% at 12 h and 75.2 and 83.6% at 24 h, respectively. According to the CCK-8 results in Figure 4B, the CBDCs on PCL/CS/SiO_2_ showed superior proliferation at days 1, 4 and 7 compared with the PCL/CS bioscaffold. A light microscopic image further confirmed that the CBDCs could adhere to the bioscaffolds on day 4 (Figure 4C–E). This result is in accordance with the adhesion experiment. The cells on the PCL/CS/SiO_2_ bioscaffold exhibited a better adherence with increased cell numbers and a better cell-fiber entanglement at 24 h, which is perhaps attributed to the enhanced surface with better hydrophilicity and more cell adhesion sites introduced by the presence of the induction factor SiO_2_ nanoparticles [42,43].

### 2.3. Osteogenic Differentiation and Evaluation

**Figure 5:**
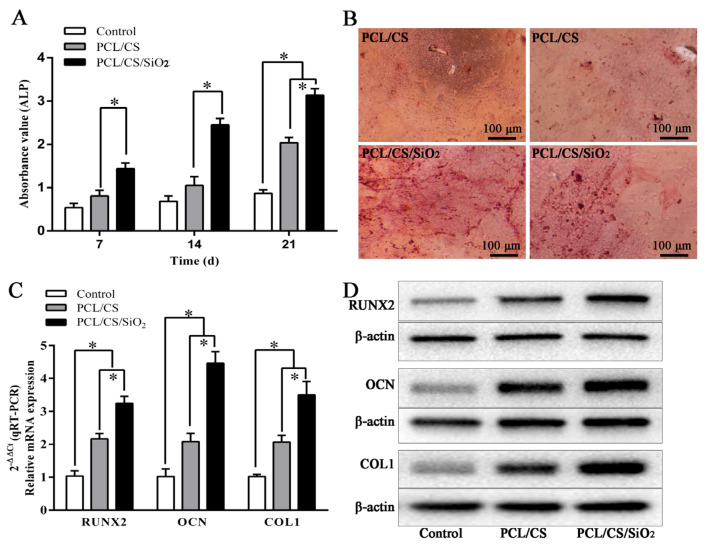
Osteogenic differentiation, the osteogenic genes and protein expression of CBDCs on the PCL/CS and PCL/CS/SiO_2_ bioscaffolds. (**A**) The ALP activity of CBDCs on the bioscaffolds (* *p* < 0.05, *n* = 3). (**B**) Calcium nodules stained on PCL/CS and PCL/CS/SiO_2_ bioscaffolds by Alizarin red at day 21, *n* = 3. (**C**) The RUNX2, OCN and COL1 gene expression of day 4 (* *p* < 0.05, *n* = 3). Expression of gene was quantified relative to that of β-actin using a sequence detector software and the relative quantification (2^−ΔΔCt^) method. (**D**) The protein expression of RUNX2, OCN and COL1 increased on the bioscaffolds on day 4, *n* = 3.

The CBDCs after 24 h seeding on the PCL/CS and PCL/CS/SiO_2_ bioscaffolds were performed for osteogenic differentiation. The osteogenic activity of the CBDCs cultured was assessed via the ALP activity (Figure 5A). On days 7, 14 and 21, the metabolic activity (OD value) of the CBDCs on the surface of the PCL/CS/SiO_2_ was higher than that on the PCL/CS bioscaffold. Alizarin red staining was used to measure the osteogenic activity and extracellular matrix (ECM) mineralization of the CBDCs in the PCL/CS and PCL/CS/SiO_2_ bioscaffolds on day 21 (Figure 5B and Figure S7). Obviously, compared with PCL/CS, the PCL/CS/SiO_2_ bioscaffold exhibited significantly better osteogenic activity with more calcium nodules stained. A microscopy investigation on the CBDCs after the osteogenic differentiation after 48 and 96 h is shown in Figure S8, indicating osteogenic differentiation. The results confirmed that by introducing the induction factor SiO_2_ into the PCL/CS bioscaffold, the osteopromoting effect was significantly improved [44].

Figure 5C shows the osteogenic genes expression evaluated after 4 days of CBDCs cultured on the PCL/CS and PCL/CS/SiO_2_ bioscaffolds. RUNX2, OCN and COL1 are key transcription factors associated with osteogenic differentiation and biomineralization. The RUNX2, OCN and COL1 gene expression increased on all the bioscaffolds on day 4 (2^−ΔΔCt^ method), and PCL/CS/SiO_2_ exhibit the maximum upregulated. Meanwhile, Figure 5D and Figure S9 displayed the protein expression of RUNX2, OCN and COL1 on the bioscaffolds on day 4. Apparently, the protein expression levels of PCL/CS/SiO_2_ are superior to the PCL/CS bioscaffold. Especially, RUNX2, which is closely related to bone formation and controls the bone growth [45], can promote the early differentiation of osteoblasts and is expressed in all stages of osteogenesis [46]. OCN plays an essential role in the differentiation of preosteoblasts into mature osteoblasts [47]. COL1 accounts for more than 90% of the bone matrix, can form a collagen matrix network binding with other proteins and can provide a settling point structure for the deposition of hydroxyapatite [47]. Thus, the superiority expression of PCL/CS/SiO_2_ achieves facilitating the bone formation of CBDCs growth.

According to the above research results, it is clear that the excellent osteogenic induction ability of the PCL/CS/SiO_2_ bioscaffold is attributed to its superior morphological and microstructure characteristics [48]. The PCL/CS bioscaffold can be used as the frame of an ECM, while the induction factor SiO_2_ nanoparticles can regulate the biological functions of CBDCs [49]. The synthesized electrospun PCL/CS/SiO_2_ bioscaffolds present a three-dimensional porous microstructure with SiO_2_ nanoparticles uniformly dispersed in the interlinked nanofibers. The electrospun porous nanofibrous structure is similar to a natural bone scaffold and is more conducive to cell growth, proliferation and migration [44]. The introduction of SiO_2_ into biopolymer nanofibers can support cell attachment, promote cell proliferation, facilitate the induction of new bone formation and integrally enhance the osteoinduction ability.

## 3. Materials and Methods

### 3.1. Materials Synthesis and Characterization

Polycaprolactone (PCL, NW = 80,000, Aladdin) and chitosan (CS, Aladdin) were purchased from Sigma-Aldrich. Tetraethoxysilane (TEOS), formic/acetic acid were bought from Sinopharm Group Chemical Co., LTD, Shanghai, China. All chemicals purchased were not further treated after receipt.

#### 3.1.1. Synthesis of PCL/CS and PCL/CS/SiO_2_ Bioscaffolds

A total of 210 mg PCL and 90 mg CS (mass ratio of 7:3) were dissolved in formic/acetic acid mixed solvent and stirred for 10 h. Electrospinning solution with 10% mass fraction was prepared and the electrospinning process was operated at a voltage of 22 kV with a flow rate of 0.2 mL h^−1^. The collected sample is denoted as PCL/CS.

SiO_2_ nanoparticles were prepared by the well-established Stöber method [50]. A certain amount of SiO_2_ nanoparticles were dispersed in the above electrospinning solution. Electrospinning process was carried out as above. The collected sample is denoted as PCL/CS/SiO_2_. For the subsequent cell measurement, the bioscaffolds were directly prepared for cell culture in the 24-well plates (Figure S10).

#### 3.1.2. Materials Characterization

The morphology, constitution and physical characteristics of PCL/CS and PCL/CS/SiO_2_ bioscaffolds were measured by simultaneous thermal analysis (TGA and DSC, TASDT650), Fourier transform infrared spectrum (FTIR, Nicolet iS50, Thermo), X-ray photoelectron spectroscopy (XPS, ESCALab250), Brunauer–Emmett–Teller (BET, 3H-2000PS1), water contact angle analysis (OCA20), optical microscope (XDS-500C), tensile property test (YG (B) 026h-500) and scanning electron microscopy (SEM, JEM-2100F). The water absorption rate was = (M_w_ − M_0_)/M_0_ × 100%, where M_w_ is absorption mass and M_0_ is initial mass. The weight loss ratio = (W_0_ − W_d_)/W_d_ × 100%, where W_0_ is the weight of the prepared samples, and W_d_ is the weight of drying at 40 °C after immersing for specified days.

### 3.2. Cell Measurement

#### 3.2.1. Preparation of Mouse CBDCs

Mouse compact bone-derived cells (CBDCs), the MSCs derived from bone cortex of the mouse, were extracted from male C57BL/6J mice (3 weeks old, Beijing Vital River Laboratory Animal Technology Co., Ltd. Beijing, China) according to the previously published procedures [51,52]. The third passage of CBDCs were used for cell measurement of the electrospun bioscaffolds (Figure S11).

#### 3.2.2. Cell Adhesion and Proliferation

CBDCs were seeded on the surface of the electrospun bioscaffolds. After 4, 12 and 24 h of cell culture, digested cells were counted to calculate the percentage of cell adhesion. The cell adhesion rate was = C/C_0_ × 100%, where C_0_ is the number of inoculated cells and C is the number of attached cells.

Cell proliferation was assessed by using the Cell Counting Kit-8 (CCK-8, Dojindo Laboratories, Kumamoto, Japan) assay. At 1, 4 and 7 days, a microplate reader was applied to measure the absorbance of solution at 450 nm to evaluate the cells’ proliferation.

#### 3.2.3. Osteogenic Induction

Alkaline phosphatase (ALP) activity assay was measured to confirm osteogenic induction. After CBDCs were seeded on the electrospun bioscaffolds for 24 h, an osteogenic induction medium was used for the following osteogenic-related assays and cells were cultured for 21 days. On days 7, 14, 21, a BCIP/NBT color development kit (Nanjing Jiancheng Bioengineering Institute, Nanjing, China) was applied for ALP staining and measured at 405 nm to investigate bone formation potentiality.

Extracellular matrix (ECM) mineralization of CBDCs on the electrospun bioscaffolds were detected by Alizarin red staining (Alizarin Red S, Solarbio, Beijing, China) at 21 days.

The total RNA of CBDCs cultured for 4 days on the bioscaffolds was extracted, the osteogenic gene expression (RUNX2, OCN and COL1) was measured by real-time PCR. The primer sequences are listed in Table S2. Cellular proteins were extracted from CBDCs on the electrospun bioscaffolds and on day 4 accessed Western blotting of osteogenic proteins as previously described methods [53]. Each sample was incubated with primary antibodies: RUNX2 (1:2000; Proteintech, Chicago, IL, USA), OCN (1:2000; Proteintech, Chicago, IL, USA), COL1 (1:1000; Proteintech, Chicago, IL, USA) and β-actin (1:5000; Proteintech, Chicago, IL, USA). The band intensities were quantitated using the Image J software V1.6.0 [54].

## 4. Conclusions

In summary, we demonstrate a novel bioscaffold for bone tissue regeneration by electrospinning PCL/CS nanofibers anchored with bioactive SiO_2_ nanoparticles. The electrospun bioscaffolds demonstrate a three-dimensional porous microstructure and make use of the virtues of PCL, CS and the osteoinductive supplement SiO_2_. The superior microstructure together with the suitable mechanical and hydrophilic properties contributed to the cell adhesion, growth, migration, proliferation and differentiation. The key feature of the bioscaffold is to make use of bioactive SiO_2_ nanoparticles to improve the osteoblast differentiation and further promote the bone induction activity. In addition, the PCL/CS/SiO_2_ bioscaffold exhibited biodegradability in a simulated body fluid environment, indicating a future trial in vivo can be expected. Thus, this work provides a novel modified bioscaffold for bone regeneration to meet the requirements for bone tissue engineering.

## Data Availability

The data presented in this study are available on request from the corresponding author.

## Supplementary Materials

The following supporting information can be downloaded at: https://www.mdpi.com/article/10.3390/molecules27248832/s1, Figure S1: The DSC heating curve of PCL/CS/SiO_2_ bioscaffold; Figure S2: The spectrum of C 1s (A) and O 1s (B) of PCL/CS/SiO_2_ bioscaffold; Figure S3: The FTIR spectra of PCL/CS and PCL/CS/SiO_2_ bioscaffolds; Figure S4: The stress–strain curves of PCL/CS and PCL/CS/SiO_2_ bioscaffolds; Figure S5: Water absorption PCL/CS and PCL/CS/SiO_2_ bioscaffolds as function of immersion time (*n* = 3); Figure S6: Weight loss ratio of PCL/CS and PCL/CS/SiO_2_ bioscaffolds as function of breakdown duration within PBS (pH = 7.4) at 37 °C (*n* = 3); Figure S7: Comparison of calcium nodules staining density after CBDCs osteogenic differentiation on the PCL/CS and PCL/CS/SiO_2_ bioscaffolds by Alizarin red at day 21, *p* < 0.05, *n* = 3; Figure S8: CBDCs after osteogenic differentiation under microscopy investigation after seeding on the bioscaffold of PCL/CS-48 h (A), PCL/CS-96 h (B), PCL/CS/SiO_2_-48 h (C) and PCL/CS/SiO_2_-96 h (D); Figure S9: Osteogenic protein expression of CBDCs on the PCL/CS and PCL/CS/SiO_2_ bioscaffolds on day 4, RUNX2 (A), OCN (B) and COL1 (C), *p* < 0.05, *n* = 3; Figure S10: The photos of the bioscaffold prepared for cell culture in the 24-well plates; Figure S11: Mouse compact bone-derived cells (CBDCs). (A) The primary culture of CBDCs. (B) The subculture of CBD1Cs; Table S1: The Brunauer–Emmett–Teller (BET) surface area, pore volume and average pore size of PCL/CS and PCL/CS/SiO_2_ bioscaffolds; Table S2: Primer sequences for osteogenic genes.

## Sample Availability

Samples of the compounds are available from the authors.
