# Supplementary material for: Nanosilica-Anchored Polycaprolactone/Chitosan Nanofibrous Bioscaffold to Boost Osteogenesis for Bone Tissue Engineering
Source: Molecules. 2022 Dec 13;27(24):8832. doi: 10.3390/molecules27248832 (PMC9786850; doi:10.3390/molecules27248832)
Supplement: Supplementary file 1 [file molecules-27-08832-s001.zip › molecules-2062533-Supplementary Materials.pdf]

Supporting information

Nanosilica-anchored Polycaprolactone/Chitosan Nanofibrous Bioscaffold to Boost Osteogenesis for Bone Tissue Engineering

Shengyou Ge [1], Xiaoyi Zhu [2], Chuanlong Zhang [2], Dongchen Jia[2], Wei Shang [1], Chao Ding[4] , Jianping Yang[3], Yuanyong Feng [1*]

[1] Department of Oral and Maxillofacial Surgery, School of Stomatology and The Affiliated Hospital of Qingdao University, No. 16 Jiangsu Road, Qingdao, 266003, China; shengyouge@163.com (S.G.); liweishang@ 126.com (W.S.)

[2] School of Environmental Science and Engineering, Qingdao University, No. 308 Ningxia Road, Qingdao 266071, China; xyzhu@qdu.edu.cn (X.Z.); qduzhangcl@163.com (C.Z.); 2020025847@qdu.edu.cn (D.J.)

[3] State Key Laboratory for Modification of Chemical Fibers and Polymer Materials, College of Materials Science and Engineering, Donghua University, Shanghai 201620, China; jianpingyang@dhu.edu.cn (J.Y.)

[4] Longkou Traditional Chinese Medicine Hospital; lkdingchao666@163.com (C.D.)

* Correspondence: feng_yuanyong@163.com (Y.F.)

Supporting Figures

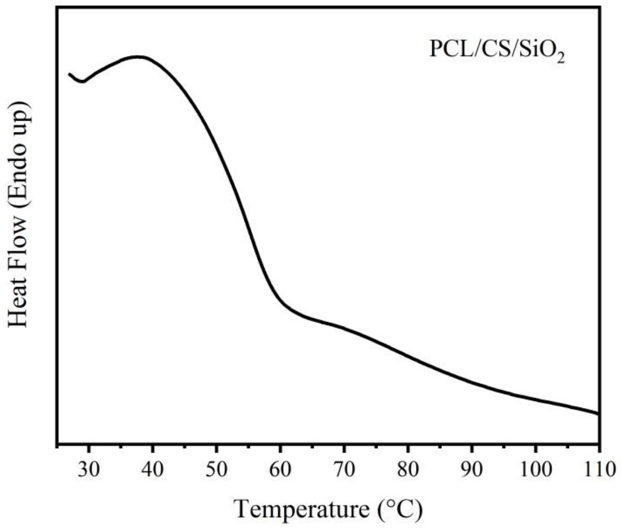

Figure S1. The DSC heating curve of PCL/CS/SiO_2 bioscaffold.

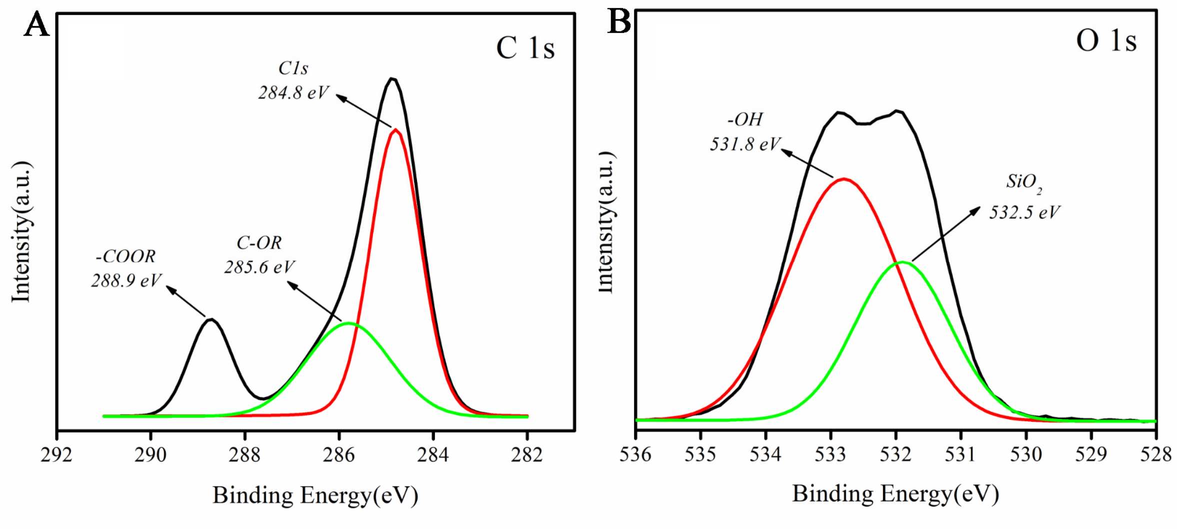

Figure S2. The spectrum of C 1s (A) and O 1s (B) of PCL/CS/SiO_2 bioscaffold.

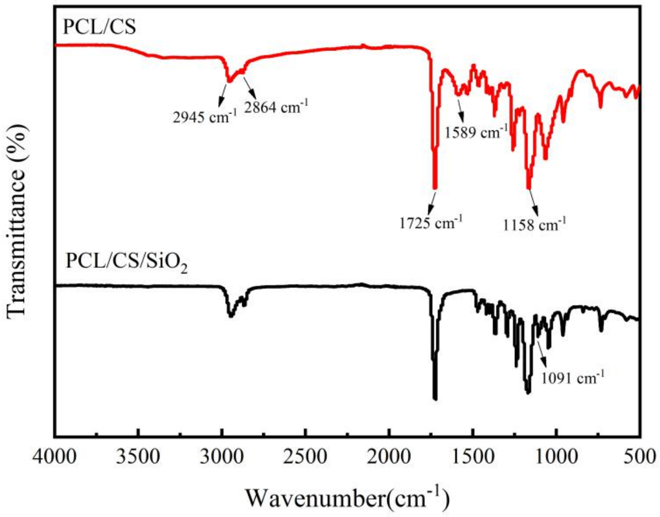

Figure S3. The FTIR spectra of PCL/CS and PCL/CS/SiO_2 bioscaffolds.

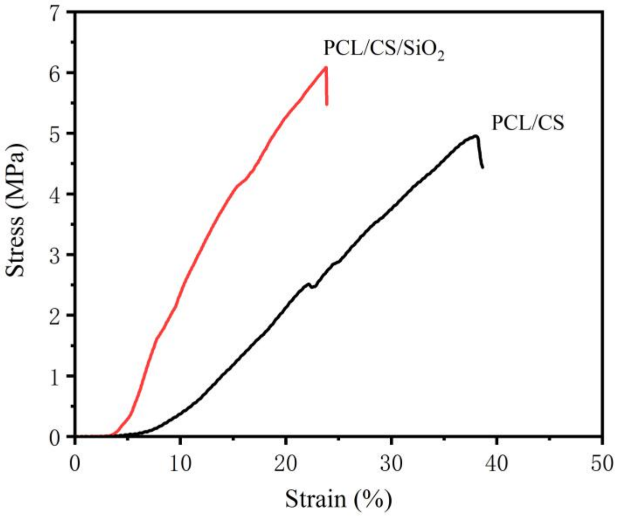

Figure S4. The stress-strain curves of pure PCL and PCL/CS/SiO_2 bioscaffold.

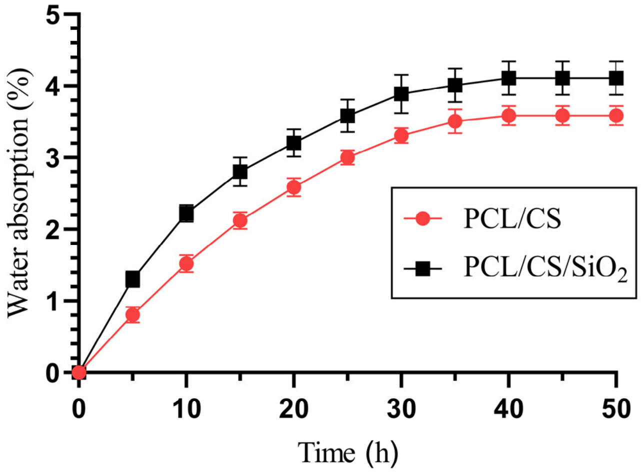

Figure S5. Water absorption PCL/CS and PCL/CS/SiO_2 bioscaffolds as function of immersion time (n = 3).

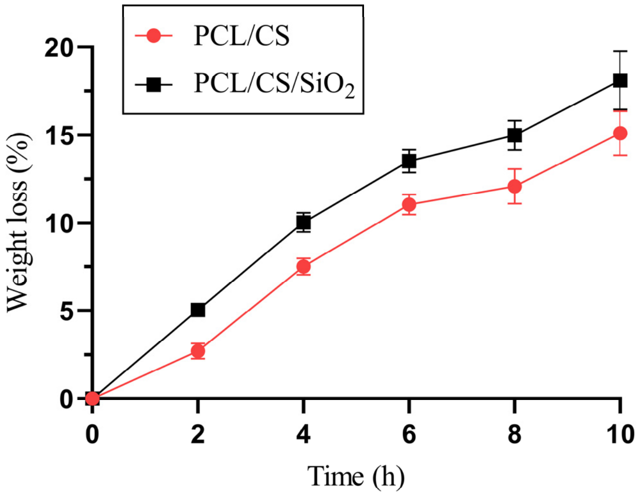

Figure S6. Weight loss ratio of PCL/CS and PCL/CS/SiO_2 bioscaffolds as function of breakdown duration within PBS (pH = 7.4) at 37 °C (n = 3).

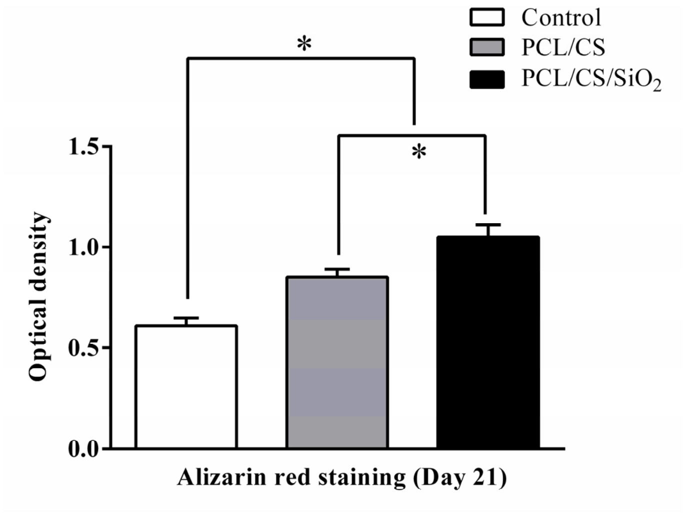

Figure S7. Comparison of calcium nodules staining density after CBDCs osteogenic differentiation on the PCL/CS and PCL/CS/SiO_2 bioscaffolds by Alizarin red at day 21, * $P < 0.05$, n = 3.

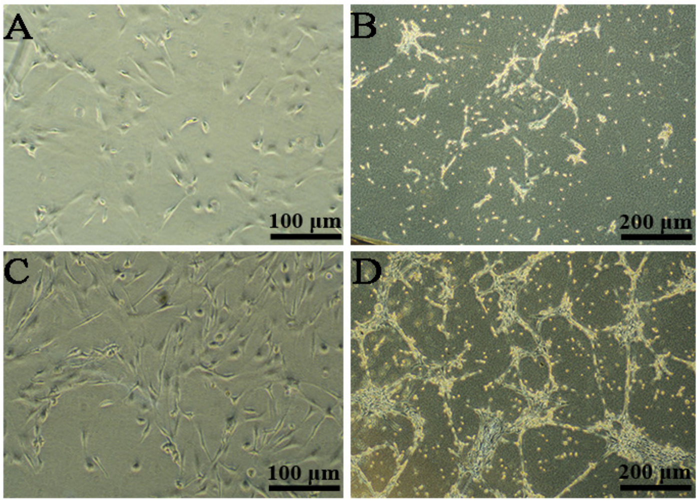

Figure S8. CBDCs after osteogenic differentiation under microscopy investigation after seeding on the bioscaffold of PCL/CS-48 h (A), PCL/CS-96 h (B), PCL/CS/SiO_2-48 h (C) and PCL/CS/SiO_2-96 h (D).

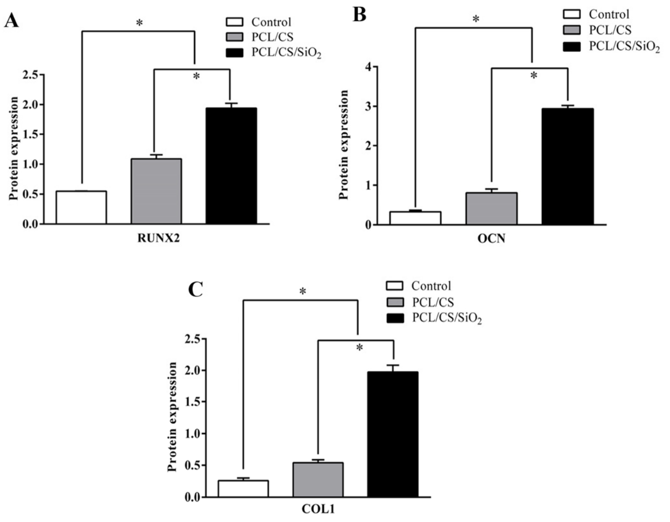

Figure S9. Osteogenic protein expression of CBDCs on the PCL/CS and $PCL/CS/SiO_2$ bioscaffoldson day 4, RUNX2 (A), OCN (B) and COL1(C) , * $P < 0.05$, n = 3.

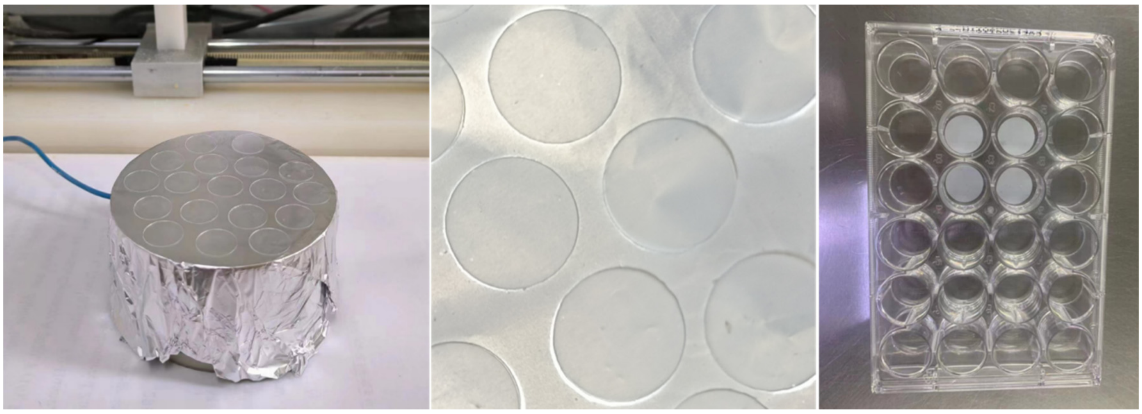

Figure S10. The photos of the bioscaffold prepared for cell culture in the 24-well plates.

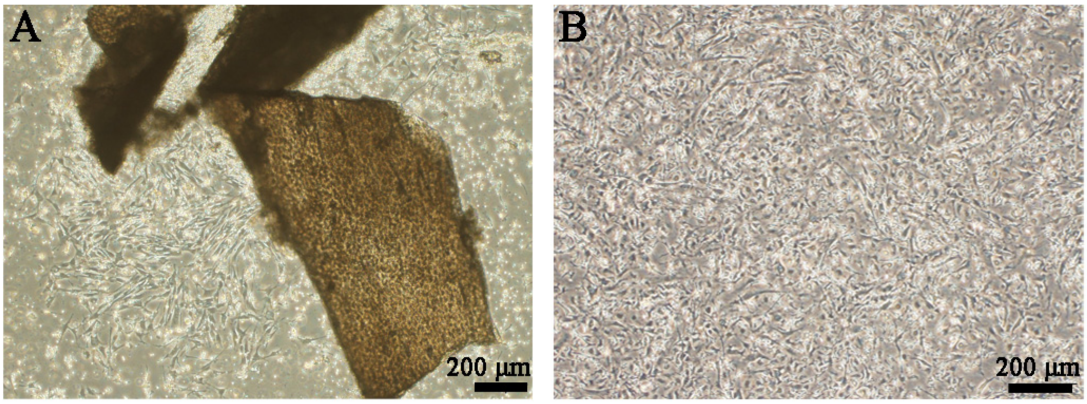

Figure S11. Mouse compact bone-derived cells (CBDCs). (A) The primary culture of CBDCs. (B) The subculture of CBDCs.

Supporting Tables

Table S1. The Brunauer-Emmett-Teller (BET) surface area, pore volume and average pore size of PCL/CS and PCL/CS/SiO_2 bioscaffolds.

Sample	PCL/CS	PCL/CS/SiO_2

**Surface area (m^2 g^{-1})**	23.94	37.68
**Pore volume (cm^3 g^{-1})**	0.05752	0.1024
**Average pore size (nm)**	9.61	10.87

Table S2. Primer sequences for osteogenic genes

Gene	Forward primers (5′- 3′)	Reverse primers (5′- 3′)
RUNX2	TCTTTTGGGATCCGAGCACC	CCGGCCCACAAATCTCAGAT
OCN	GGAGCCTTGCTACCGTTCAT	GCCTTCACTGATACGCGTTC
COL1	TCTTACAGTTCCACGGCCAC	AGCATCTGAGCTGGAGGGTA
GAPDH	TTCACCACCATGGAGAAGGC	TGAAGTCGCAGGAGACAACC
